# Perception of the HACCP system operators on livestock product manufacturers

**DOI:** 10.1186/2055-0391-56-19

**Published:** 2014-09-24

**Authors:** Jung-Hyun Kim, Ki-Chang Nam, Cheorun Jo, Dong-Gyun Lim

**Affiliations:** Department of Public Health, Graduate School of Public Health, Seoul National University, Seoul, 151-742 Korea; Department of Animal Science and Technology, Sunchon National University, Suncheon, 540-742 Korea; Department of Agricultural Biotechnology and Research Institute of Agriculture and Life Science, Seoul National University, Seoul, 151-921 Korea; Department of Health Administration and Food Hygiene, Jinju Health College, Jinju, 660-757 Korea

**Keywords:** HACCP system operator, Livestock product plants, Survey, Monitoring, Prospect of occupation

## Abstract

The purpose of this study was to investigate crucial factors on HACCP system implementation in domestic livestock product plants, and to offer job satisfaction and the career prospect of HACCP system operators. The survey was carried out by selecting 150 HACCP system operators who implemented HACCP system. The respondents claimed that the most important contents in HACCP system operation were to assemble HACCP team (21.8%), and the second was to monitoring (20.0%). Documentation and recording (16.9%) and verification (11.1%) were followed. The respondents answered the major factor in sanitation management was cleaning/washing/disinfection (18.9%) and inspection (18.4%). The results showed that there were significant differences in the prospect of occupation in HACCP system operator by the gender (p < 0.015), age, livestock product facilities, service period, and position (p < 0.001). The respondents from HACCP system operator were satisfied with their job (73%) and also showed optimistic prospect of occupation (82%).

## Background

The safety of food products has become a major issue of concern. Hazard analysis critical control points (HACCP) is a food safety management system [1], widely acknowledged as the best method of assuring product safety while becoming internationally recognized as a tool for controlling food borne safety hazards (Codex Alimentarius Commission, [2, 3]). HACCP is a systematic approach to the identification, evaluation, and control of hazards in those steps in food manufacturing that is critical to food safety (Ropkins et al., [4]).

In several countries, including Korea, hazard analysis critical control point (HACCP) systems have been introduced with regard to product hygiene and safety (Codex Alimentarius Commission, [5]). In Korea, regulatory authorities have introduced HACCP systems on meat processing plant in 1997, slaughter house in 2000, livestock product plant in 2001, milk processing plant, meat sale and distribution in 2004, feed mill in 2005, and animal farm in 2006 [6]. HACCP implementation of the slaughterhouse in livestock product field is only mandatory in Korea. The HACCP system is being increasingly used in many food industries under regulatory agencies. Most developed countries including USA (1998), EU (1996) and Australia (1997) are implementing HACCP system as an obligation.

It is doubtful if any company can implement HACCP without trained-HACCP team members. This is particularly true for the small-scale company with limited access to information [7]. Competency in HACCP can be effectively gained through training and this must be complemented with the appropriate knowledge of food sanitation and food microbiology. Research has observed that the employment of an experienced, technically qualified person is the single most important factor influencing the implementation of HACCP [8].

The studies on HACCP have recently focused on evaluation of sanitation management performance, benefits of HACCP implementation, and employees' knowledge and performance degree of HACCP in school foodservice sector [9–11]. In the livestock products sector, economic feasibility of HACCP at slaughterhouse [12], and comparative analysis of the prerequisite items for HACCP in livestock product plants [13] have been reported. However, there has not yet been studied about the basic information for job order of priority and career prospect of HACCP system operators (HACCP and microbiological analysis operator) in livestock products sector. Therefore, the objective of this study was not only to investigate crucial factors on HACCP system implementation in domestic livestock product plants, but also to offer job satisfaction and the career prospect of HACCP system operators in livestock product industry.

## Methods

### Study population

This study was based on data obtained 15 livestock product manufacturer (5 large scale plants, 10 medium scale plants) located in Korea. A survey was conducted with subjects to operate HACCP in livestock product plants. Most of the respondents were HACCP team members (HACCP and microbiological analysis operators) who are in charge of quality control department of the manufacturer. The survey was carried out with 150 respondents who implemented HACCP system. The survey questionnaire was performed by some questions with multiple choices for the answers and consisted of 11 questions including 7 questions of general characteristics, 2 questions of priority of duties in HACCP system operation in plants and 2 questions of a job analysis.

### Description of demographic variables

In the study, demographic variable included gender, age, educational and income level, size of livestock product facilities, service period, and position. Age reported at the time of interview was categorized into three groups: less than 29, 30-39, and 40-49. Educational level was divided into college and university. Income level per month was categorized into four groups: less than 200, 200 to 250, 250 to 300, and more than 300 Korean won. Livestock product facilities were divided into two categories: small- or medium and large-scale. The service period was classified into three groups: less than 1, 3-5, and 5-10 years. Position was categorized into four groups: employees, team manager, assistant manager, and president, director. All participants provided written and informed consent to participate in the study. Respondents were individually given adequate time to answer each query in writing.

### Statistical analysis

Statistical analysis was performed using the STATA software (version12.0) for Window and Two-sided P-value <0.05 was considered statistically significant. General characteristics of the study participants and priority of duties in HACCP system operation and sanitation management are presented as frequency and percentage. The chi-square tests were performed to investigate the differences among job satisfaction and prospect of occupation in HACCP system operators by age, educational and income level, size of food product facilities, career, and position.

## Results and discussion

General characteristics of the study participants are shown in Table 1. Overall, male subjects are 68% and female ones are 32%. Among age variable, age is 20-29 (46.7%), 30-39 (33.3%), and 40-49 (20%) respectively. In educational level, the respondents have acquired a college diploma (58.7%) or university degree (41.3%). Income level per month is under the 3 million won (35.3%), 2 million won (29.3%), 2.5 million won (25.3%), and over 3 million won (10.0%). Size of livestock product facilities is; small and medium-scale facilities were mostly 73.3% and large-scale ones were respectively 26.7%. Career duration is; the highest was less than 1 year (56.7%), whereas 3-5 year was the lowest (8.7%) under a year workers have been working as a current HACCP system operators in manufactures. The position is employees (53.3%), team manager (28%), assistant manager (17.3%) and president (CEO) (1.3%).

**Table 1:** **General characteristics of respondents participated (N = 150)**

Characteristics		Frequency (N)	Percent (%)
Gender	Male	102	68
	Female	48	32
Age	20-29	70	46.7
	30-39	50	33.3
	40-49	30	20.0
Educational background	≥College diploma	88	58.7
	University degree	62	41.3
Income per month (million won)	<2	44	29.3
	<2.5	38	25.3
	<3	53	35.3
	≥3	15	10.0
Size of processing facilities	Large-scale	40	26.7
	Small and medium-scale	110	73.3
Service period (yrs.)	<1	85	56.7
	3-5	13	8.7
	5-10	52	34.7
Position	Employees	2	53.3
	Team manager	42	28
	Assistant manager	26	17.3
	President, CEO	80	1.3
Total		150	100

Table 2 shows the priority of duties in HACCP system operation in plants. Basically, HACCP system is a science-based system created to identify specific hazards and actions to control them in order to ensure food safety. It is also a systematic process: a sequence of twelve tasks has been described, in which the seven basic HACCP principles are included (Codex Alimentarius Commission, [14]). The respondents claimed that the most important contents in HACCP system operation were to assemble HACCP team (21.8%), and the second was to monitoring (20.0%). Documentation and recording (16.9%) and verification (11.1%) were followed. The reason for choosing first on assembling HACCP team might be due to the size of food companies. While relatively large-scale manufacturer will find it easier to find human resource and technical assistance, small and medium-scale businesses find it more difficult because they lack appropriate human resources, technical knowledge and experience to introduce HACCP into practice. Particularly, small food processors tend to employ the staff they need to carry out production tasks, to think only in terms of productivity rather than safety and to understand the HACCP system as complicated and unnecessary to produce food products. Therefore, the introduction of HACCP into these companies is more difficult than in large ones to use HACCP [15, 16]. It is confirmed that small-scale ones were less likely to invest in hygiene and food safety than larger ones [17]. Thus, HACCP operator with the ability to manage if the HACCP system is working correctively is urgently required.

**Table 2:** **Priority of duties in HACCP system operation in plants**

HACCP system operation	Frequency (N)	Percent (%)
Assemble HACCP team	98	21.8
Monitoring	90	20.0
Documentation and recording	76	16.9
Verification	50	11.1
Establish a corrective actions	29	6.4
Establish critical limits for each CCP	28	6.2
Describe product and identify intended use	27	6.0
Construct flow diagram and on-site confirmation of it	20	4.4
Conduct a Hazard Analysis	17	3.8
CCP determination	15	3.3
Total	450	100

Monitoring in HACCP means checking that the preventative measure at a CCP is under control to prevent hazard [18]. Monitoring was the second selected reason (20.0%) for the implementation of the HACCP system. The third most frequent given reason was a documentation and recording (16.9%). Records related to steps and procedures of HACCP must be fully completed and signed by responsible person, which adds an extra task to the routine work of food processing. Managers and staff, particularly in small businesses, require a great deal of paper work [19]. Verification was the fourth selected reason (11.1%) for the implementation of the HACCP system. The Codex Alimentarius defines verification as the application of methods, procedures, tests and other evaluations in addition to monitoring to determine compliance with the HACCP plan [19]. As stated in the sixth principles, verification includes all activities (e. g. auditing, food analysis and test), which are focused on determining that all health hazards are controlled [19]. As mentioned in Table 2, 4.4% reported to have a constructing flow diagram and on-site confirmation of it. Food factory layout must be designed to achieve a smooth flow of operations keeping the amount of handling of food materials to the minimum possible.

The World Health Organization has published a definition for prerequisites (WHO, [20]) “practices and conditions needed prior to and during the implementation of HACCP and which are essential for food safety” and again mentions that these are described in Codex Alimentairus Commission’s General Principles of Food Hygiene and other Codes of Practice. The concepts of prerequisite program (PRP) and how it will benefit HACCP had been reported by Wallace and Williams [21]. It has been recommended that before HACCP is utilized, a prerequisite program is needed [22].

Table 3 lists the priority of duties in sanitation management related to pre-requisites programs in plants. Sanitation management consisted of 11 tasks, where the importance of the field is hygiene control. Duties of importance are like these; cleaning/washing/disinfection (18.9%), inspection (18.4%), water supply (16.4%), pest control (10.9%), and employee hygiene (7.8%). It can be quite affected an entire sanitation management such as cleaning, washing and sterilizing; cause by neglecting the management of hygiene. For example, it could be caused germs; dirt hands, a rust knife, cutting board and bacterial pollution. That is, it is thought that they were required to have high level of sanitary duties; keeping a clean knife, sterilizing cutting board, and washing hands for the final product of the process. Equipment should also be designed and constructed so that cleaning, maintenance and inspection are facilitated. Well designed and structured premises with reliable equipment could help in maintaining hygienic conditions, improving cleanliness and cleaning effectiveness and controlling pest infestations [23]. However, food premises with congested and unhygienically designed food preparation rooms are frequently found. Normally, this is the case in small businesses that have been increasing their productivity without the consequent expansion of their facilities and installations, or businesses that are crowded with staff and machinery to satisfy workloads. In those situations, the implementation of HACCP is far more complicated due to the difficulty of controlling basic sanitary standards [19].

**Table 3:** **Priority of duties in sanitation management in plants**

Sanitation management	Frequency (N)	Percent (%)
Cleaning/Washing/disinfection	85	18.9
Inspection	83	18.4
Water supply	74	16.4
Pest control	49	10.9
Employee hygiene	35	7.8
Ventilation	34	7.6
Record of receiving raw materials	29	6.4
Facility and equipment	28	6.2
Transportation	16	3.6
Recall	13	2.9
Storage	4	0.9
Total	450	100

Our results suggest that food product plants in Korea were more likely to implement HACCP to improve hygiene ability rather than for other reasons. This result might be related with some reasons. First, Korean consumers showed increased the knowledge about food hygiene result from bovine spongiform encephalopathy (BSE), foot and mouth disease (FMD), and avian Influenza (AI) etc. Secondly, food hygiene might be the most important factors for livestock product plants employers and employees because SSOP was compulsorily applied for food products processing plant in Korea.

Table 4 represents the job satisfaction in HACCP system operators by gender, age, size of food product facilities, service period, and position. Approximately 83% of respondents indicated that they were satisfied with their jobs. Overall, both male and female were satisfied with their jobs. The proportion of male who thought of themselves as “Agree” in job satisfaction is higher than that of female. Concerning the age, the highest proportion of “Agree” was the age group 40-49 (100%) whereas the highest that of “Disagree” was the age group 20-29 (15.7%); however the job satisfaction increased with linearly age. The proportion of workers at large scale company who thought of themselves as “Agree” in is higher than that of small scale one. Regarding service period, there was a U-shaped association between service period and job satisfaction although there was no significant difference across the service period. The highest proportion of “Agree” by position was team manager and over (100.0%), whereas employees were the lowest (13.8%). The Chi-square test showed that the gender, age, livestock product facilities, and position significantly affected to job satisfaction (p < 0.001). The respondents showed an optimistic attitude on job satisfaction as HACCP system operator.

**Table 4:** **Job satisfaction in HACCP system operators by gender, age, size of facilities, service period, and position**

		Disagree (%)	Moderate (%)	Agree (%)	Total (N)	_x_^2^	df	***p***
Gender	Male	11(10.8)	6(5.9)	85(83.3)	102	39.353	2	0.000
	Female	0(0)	23(47.9)	25(52.1)	48			
	Total	11(7.3)	29(19.3)	110(73.3)	150			
Age (yrs)	20-29	11(15.7)	15(21.4)	44(62.9)	70	24.442	4	0.000
	30-39	0(0)	14(28.0)	36(72.0)	50			
	40-49	0(0)	0(0)	30(100.0)	30			
	Total	11(7.3)	29(19.3)	110(73.3)	150			
Size of facilities	Large-scale	0(0)	0(0)	40(100.0)	40	19.835	2	0.000
	Small-scale	11(10.0)	29(26.4)	70(63.6)	110			
	Total	11(7.3)	29(19.3)	110(73.3)	150			
Service period (yrs.)	<1	11(12.9)	15(17.6)	59(69.4)	85	14.039	4	0.007
	3-5	0(0)	0(0)	13(100.0)	13			
	5-10	0(0)	14(26.9)	38(73.1)	52			
	Total	11(7.3)	29(19.3)	110(73.3)	150			
Position	President, Director	0(0)	0(0)	2(100.0)	2	42.727	6	0.000
	Assistant manager	0(0)	0(0)	42(100.0)	42			
	Team manager	0(0)	0(0)	26(100.0)	26			
	Employees	11(13.8)	29(36.3)	40(50.0)	80			
	Total	11(7.3)	29(19.3)	110(73.3)	150			

Table 5 depicts the prospect of occupation in HACCP system operators by gender, age, size of food product facilities, service period, and position. The same trends shown in Table 4 were observed except for service period. A total of 82% of respondents showed optimistic prospect of occupation. Subjects aged 20-29 and 40-49 reported that HACCP system operator would be hopeful, whereas those aged 30-39 thought of the same as now. Furthermore, it was higher than those aged 20-29 and 40-49. The proportion of workers at large scale who thought of themselves as “Agree” in is higher than that of small scale. Regarding service period, the lowest proportion of “Agree” by career was 5-10 years group and over (48.1%), whereas less than 1 and 3-5 years group were the highest (100.0%). Especially, the prospect of occupation in HACCP system operator decreased with service period and there was significant difference across service period. The results of Chi-square testing showed that there were significant differences in the prospect of occupation in HACCP system operator by the gender (p < 0.015), age, livestock product facilities, service period, and position (p < 0.001).

**Table 5:** **Prospect of occupation in HACCP system operators by gender, age, size of facilities, service period, and position**

		Moderate (%)	Agree (%)	Total (N)	_x_^2^	df	***p***
Gender	Male	13(12.7)	89(87.3)	102	5.963	1	0.015
	Female	14(29.2)	34(70.8)	48			
	Total	27(18.0)	123(82.0)	150			
Age (yrs)	20-29	0(0)	70(100.0)	70	24.442	4	0.000
	30-39	227(54.0)	23(46.0)	50			
	40-49	0(0)	30(100.0)	30			
	Total	27(18.0)	123(82.0)	150			
Size of facilities	Large-scale	0(0)	40(100.0)	40	11.973	1	0.001
	Small-scale	27(24.5)	83(75.5)	110			
	Total	27(18.0)	123(82.0)	150			
Service period (yrs.)	<1	0(0)	85(100.0)	85	62.054	2	0.000
	3-5	0(0)	13(100.0)	13			
	5-10	27(51.9)	25(48.1)	52			
	Total	27(18.0)	123(82.0)	150			
Position	President, Director	0(0)	2(100.0)	2	27.710	3	0.000
	Assistant manager	0(0)	4(100.0)	4(100.0)			
	Team manager	13(50.0)	13(50.0)	26(100.0)			
	Employees	14(17.5)	66(82.5)	80(100.0)			
	Total	27(18.0)	123(82.0)	150			

The main role of HACCP system operators is to encourage and motivate supervisory staff and food handlers on different aspects of the HACCP concept. Consequently, they will need to attribute responsibilities between personnel involved with the implementation of the system. This must be done in accordance to the difficulty of the operation and the capabilities of the person who is going to be responsible for it [19].

## Conclusions

In conclusion, most of respondents answered an optimistic attitude on job satisfaction as HACCP system operator. The respondents from HACCP system operator were also showed optimistic prospect of occupation. Results from this study could be used to better educate HACCP system operators and industry implementers.
